# Supplementary material for: Systematic design of superaerophobic nanotube-array electrode comprised of transition-metal sulfides for overall water splitting
Source: Nat Commun. 2018 Jun 22;9:2452. doi: 10.1038/s41467-018-04888-0 (PMC6014975; doi:10.1038/s41467-018-04888-0)
Supplement: Supplementary file 1 — Supplementary Information [file 41467_2018_4888_MOESM1_ESM.pdf]

Systematic design of superaerophobic nanotube-array electrode comprised of transition-metal sulfides for overall water splitting

Haoyi Li[1]*, Shuangming Chen*[2]*, Ying Zhang*[3]*, Qinghua Zhang*[4]*, Xiaofan Jia*[5]*, Qi Zhang*[1]*, Lin Gu*[4]*, Xiaoming Sun*[3]*, Li Song*[2] *and Xun Wang*[1]

Supplementary Figures

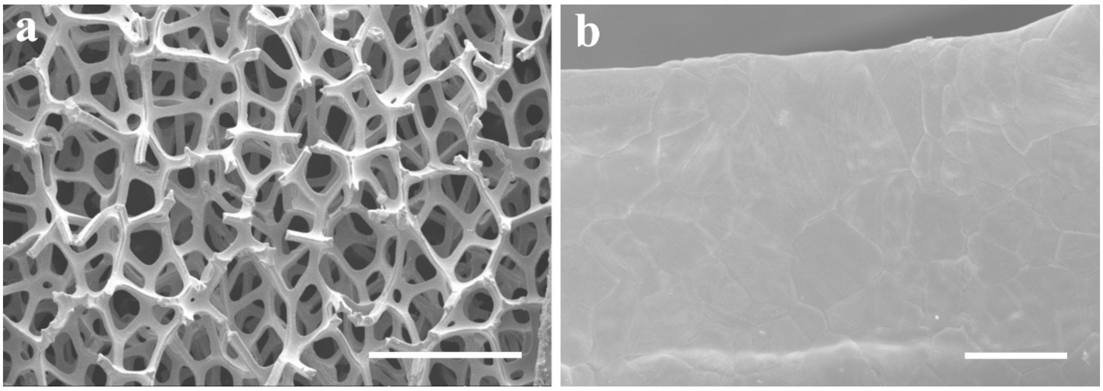

Supplementary Figure 1. The morphology of Ni foam. a,b, FESEM images of Ni foam. Scale bar, **a,** 1 mm; **b,** 20 μm.

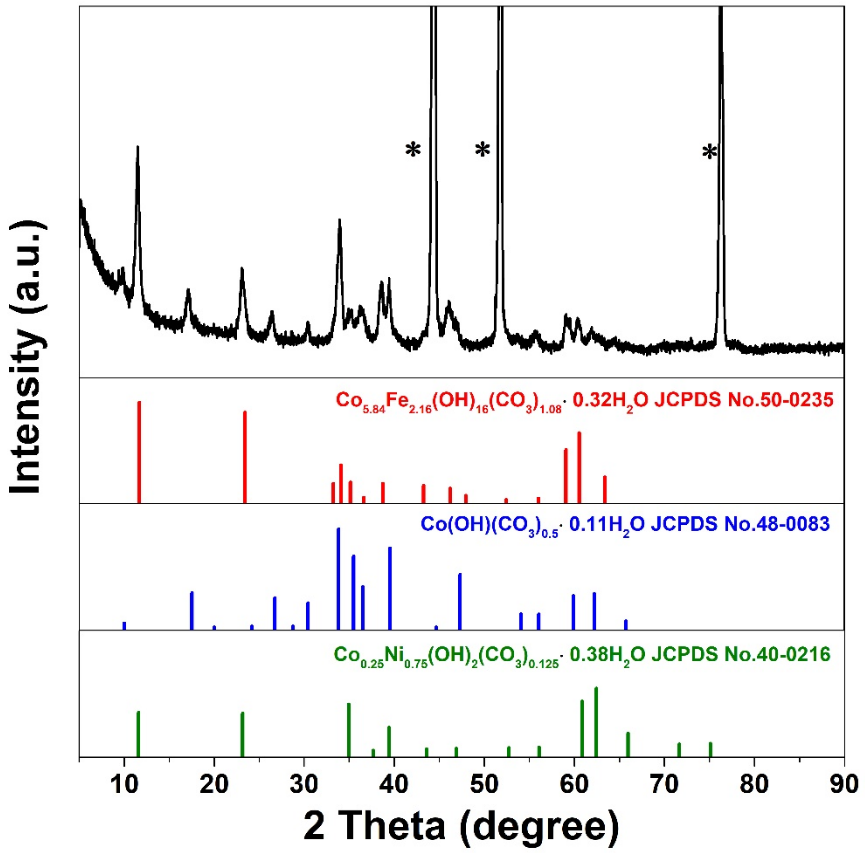

Supplementary Figure 2. The crystalline structures of FeCoNi-LDH-NWAs. XRD pattern of FeCoNi-LDH-NWAs. The red lines present the standard cobalt iron carbonate hydroxide (JCPDS No. 50-0235) peaks, the blue lines demonstrate the standard cobalt carbonate hydroxide (JCPDS No. 48-0083) peaks and the olive lines display the standard cobalt nickel carbonate hydroxide (JCPDS No. 40-0216) peaks. The three peaks of the sample marked by stars represent nickel metal from the Ni foam.

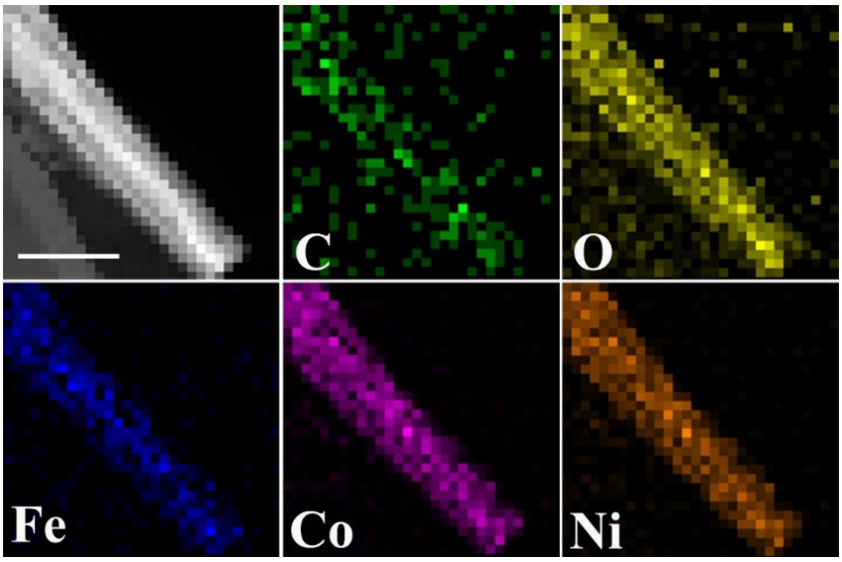

Supplementary Figure 3. The elemental composition of FeCoNi-LDH-NWAs. STEM and EDX mapping spectra of FeCoNi-LDH-NWAs. Scale bars: 200 nm. The five elements, Fe (blue), Co (pink), Ni (orange), C (green) and O (yellow), uniformly distribute in a single ternary LDH nanowire.

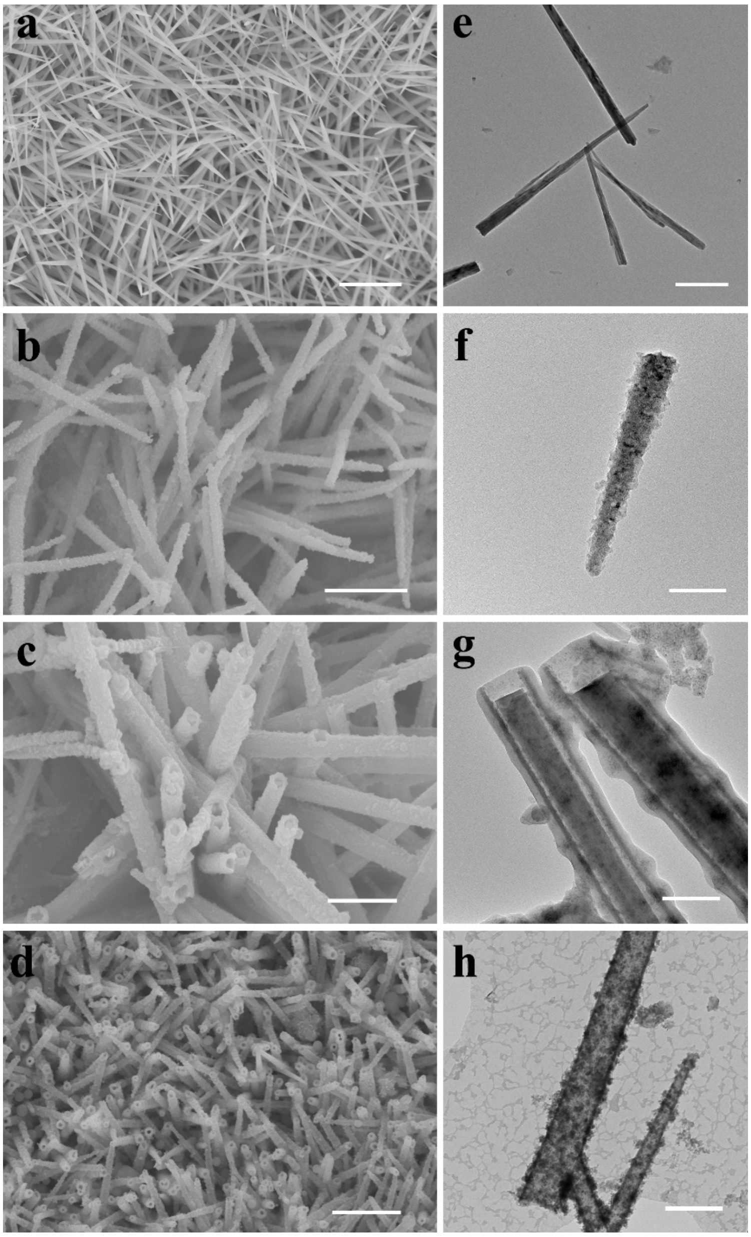

Supplementary Figure 4. The synthetic mechanism investigation of FeCoNi-HNTAs. FESEM and TEM images of the as-prepared **a**,**e**, FeCoNi-LDH-NWAs and products obtained at different reaction time: **b**,**f**, 2 h; **c**,**g**, 6 h; **d**,**h**, 10 h. Scale bar: **a**,**b**, **d**, 2 μm; **c**, 1 μm; **e**, 500 nm; **f-h**, 200 nm. Based on the as-prepared FeCoNi-LDH-NWAs, MoS_2 nanosheets grew on their walls in the beginning of the whole reaction. The obtained hybrid nanowires (FeCoNi-HNWAs) were still solid but uneven on the surface. When prolonging the reaction time to 6 h, fascinating core-shell nanostructures could be collected, which was attributed to the different diffusion rates between outward process of Fe, Co, Ni-based part and inward process of MoS_2 part. As the reaction was coming to an end, Kirkendall voids formed and well-defined nanotubes could be obtained. It is referentially significant to construct hybrid hollow nanostructures via Kirkendall cavitation using MoS_2 nanosheets as the guest through such a strategy.

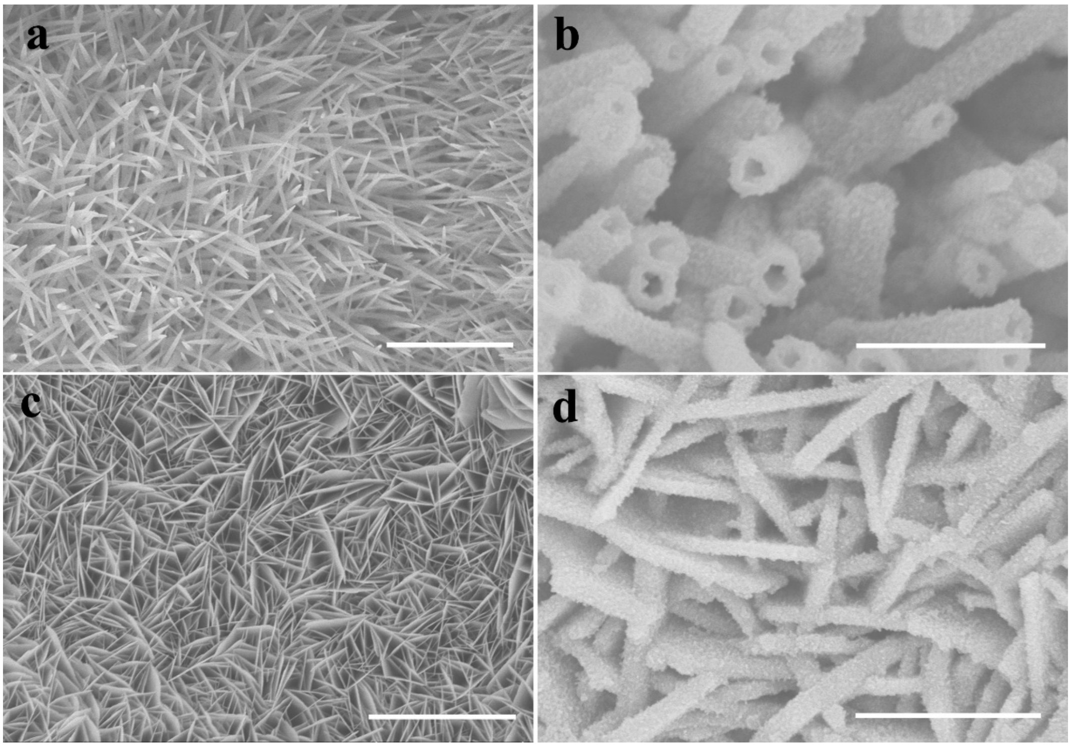

Supplementary Figure 5. The universality of the synthetic strategy. a-d, FESEM images of CoNi-LDH-NWAs, CoNi-HNTAs, NiFe-LDH-NSAs and NiFe-HNSAs, respectively. Scale bar: **a**, 5 μm; **b**, 1 μm; **c**, 10 μm; **d**, 2 μm. As the expansion of this method to synthesize MoS_2-based hybrid nanoarrays, we also used it to manufacture two other hybrid nanoarrays. The corresponding bimetal LDH nanoarrays, CoNi-LDH-NWAs and NiFe-LDH-NSAs, were used as precursors and templates to synthesize CoNi-HNTAs and NiFe-HNSAs. The two sorts of hybrid nanoarrays show uniform morphologies and uneven surface, verifying the universality of this method. On the other hand, the above results prove that Co ions play a key role in the evolution of one-dimensional nanowire morphology, decreasing the size of nanoarrays to provide more effective surface areas, which is in favor of electrocatalytic activity enhancement.

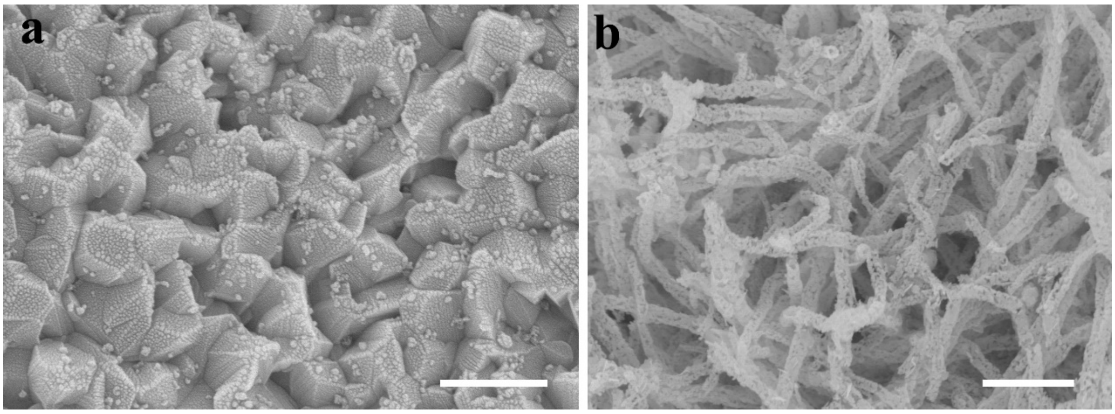

Supplementary Figure 6. The morphologies of contrastive samples. a,b, FESEM images of MoS_2/Ni Foam and FeCoNiS-NTAs. Scale bar: 1 μm.

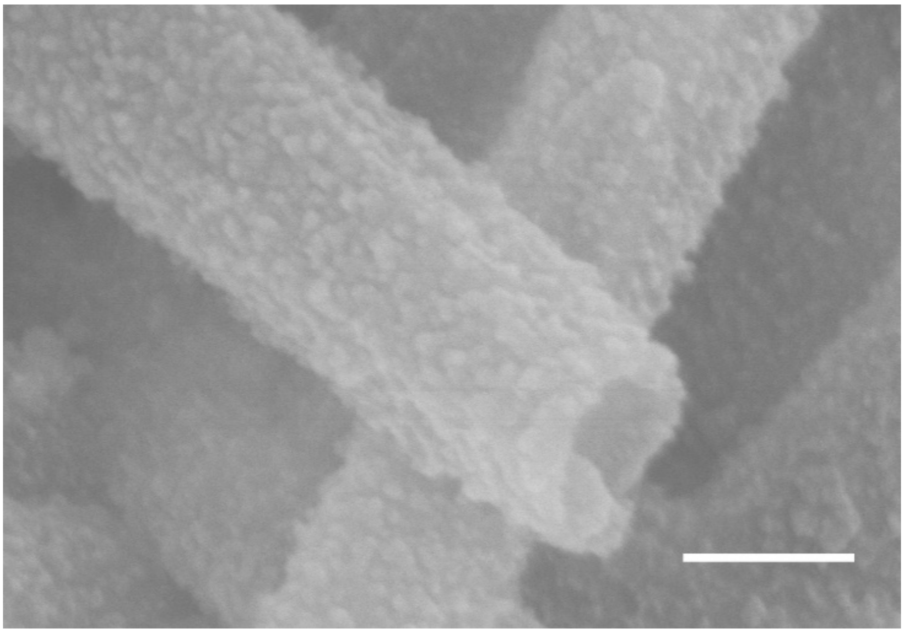

Supplementary Figure 7. The morphology of a single nanotube in FeCoNi-HNTAs. FESEM image of a single nanotube in FeCoNi-HNTAs showing the hollow and rough surface features. Scale bar: 100 nm.

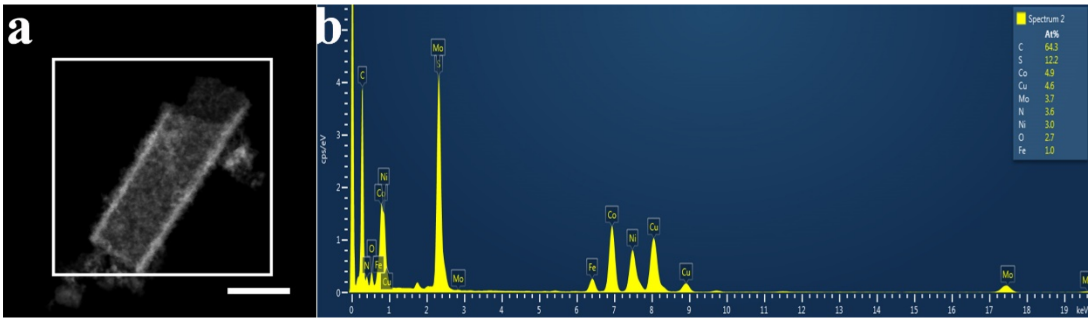

Supplementary Figure 8. The elemental ratios in FeCoNi-HNTAs. a, STEM image of FeCoNi-HNTAs. Scale bar, 100 nm. **b**, EDX spectra of the area marked in **a**.

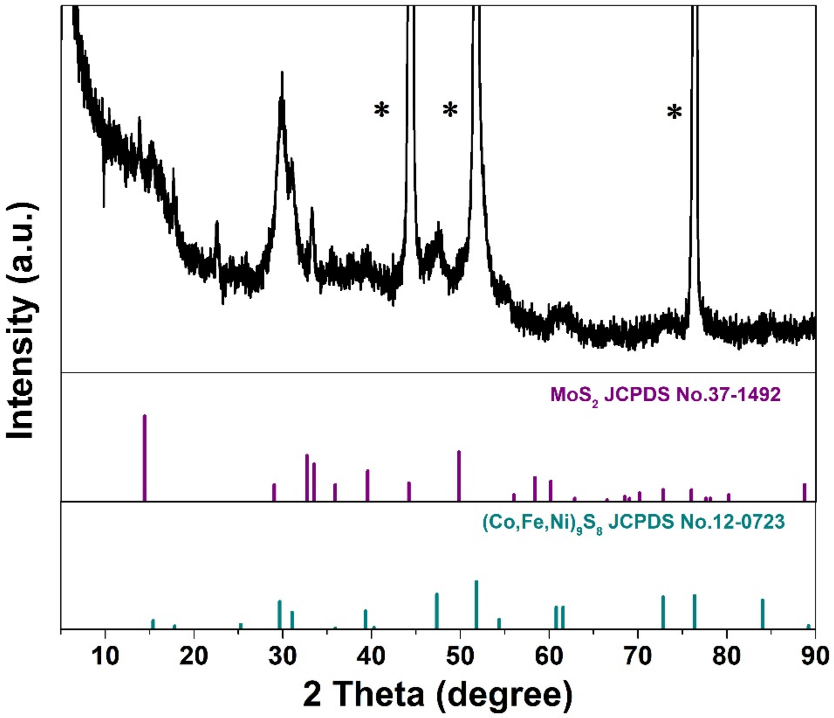

Supplementary Figure 9. The crystalline structures of FeCoNi-HNTAs. XRD pattern of FeCoNi-HNTAs exhibiting the successful formation of desired hybrid structure. The purple lines present the standard MoS_2 (JCPDS No.37-1492) peaks and the dark cyan lines demonstrate the standard $(Co,Fe,Ni)_9S_8$ (JCPDS No.12-0723) peaks, which are matched well with the experimental peaks of FeCoNi-HNTAs. The three peaks of the sample marked by stars represent nickel metal from the Ni foam.

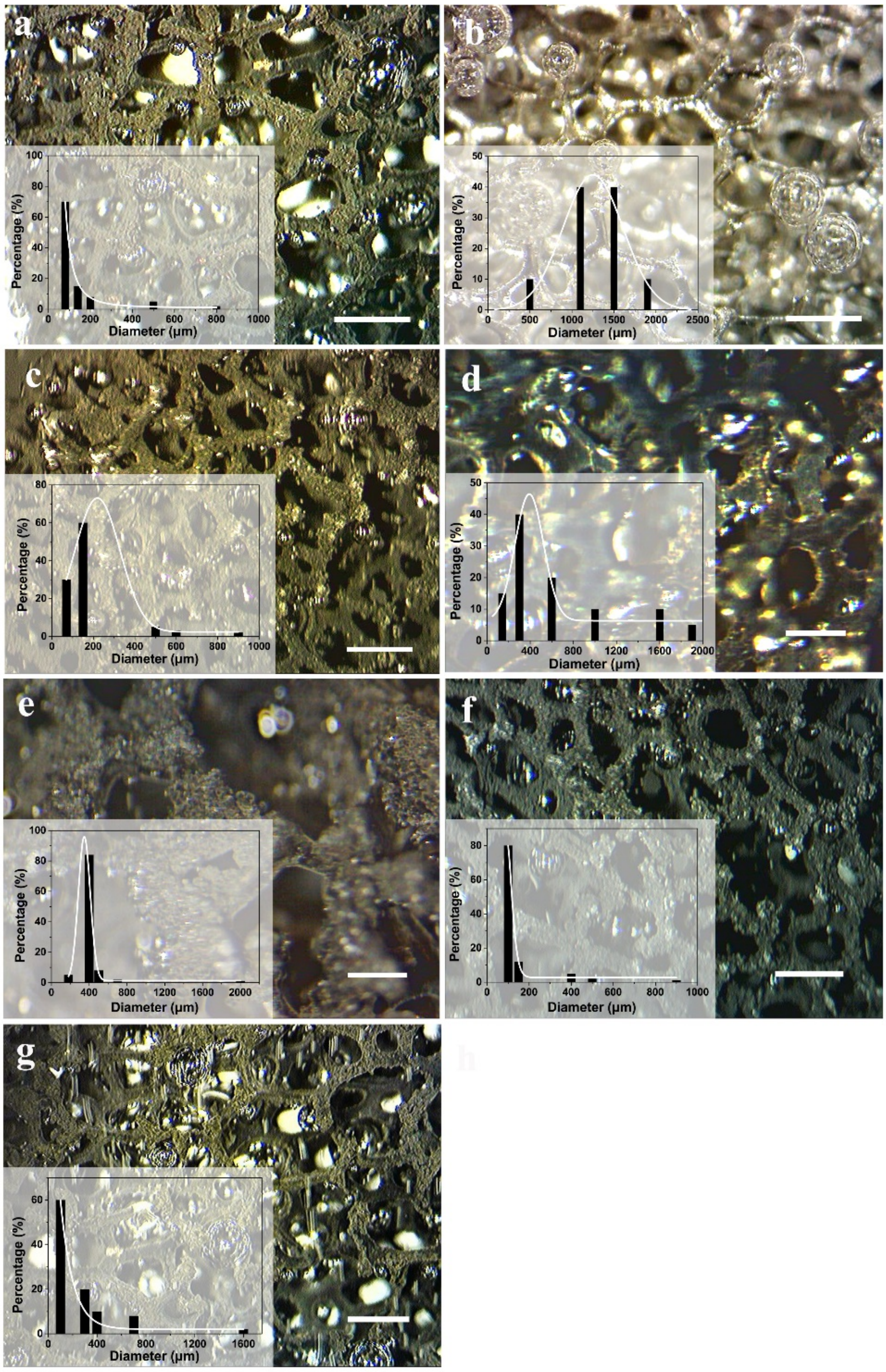

Supplementary Figure 10. The bubble releasing behaviours. Digital photos showing the as-formed bubbles releasing behaviors on the surface of **a**, FeCoNi-HNTAs; **b**, bare Ni foam and **c**, FeCoNi-LDH-NWAs for OER; **d**,**e**, and **f,g,** MoS_2/Ni Foam and FeCoNiS-NTAs for HER and OER respectively. Scale bar: 2 mm. Insets are the corresponding statistics of size distribution of releasing bubbles. These results visually exhibit that the size of releasing bubbles from nanoarray electrodes is much smaller than that of non-nanoarray electrodes in diameter, benefitting for the sufficient contact between electrode and electrolyte and decreases the “dead area”,

deterred active sites, resulting in the promotion of mass transfer.

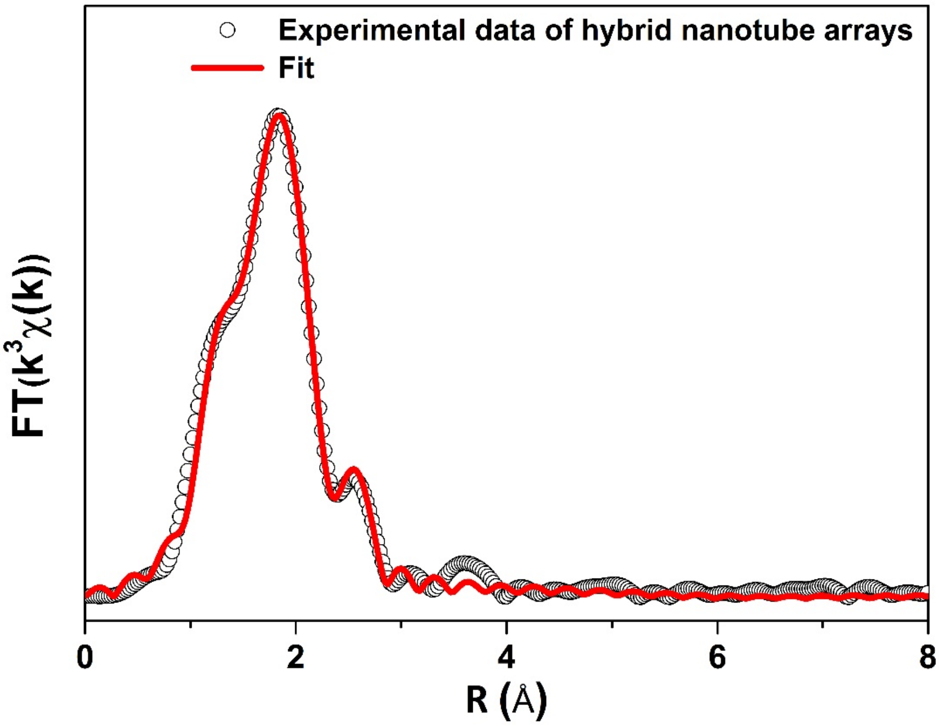

Supplementary Figure 11. The fitting data of EXAFS. Comparison between experimental data and the fitting curves of MoS_2 in FeCoNi-HNTAs for EXAFS at Mo K-edge.

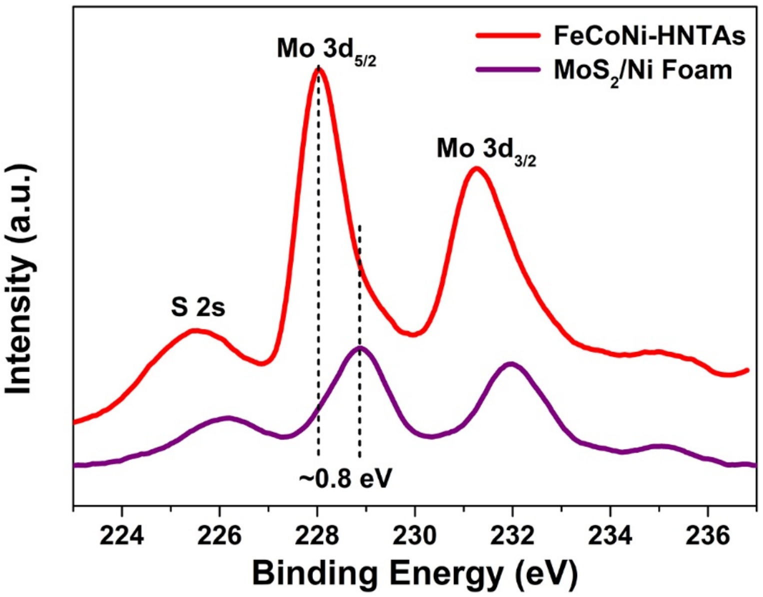

Supplementary Figure 12. The synergistic effect between the two compositions in FeCoNi-HNTAs. High-resolution XPS spectra comparison of Mo 3d regions between FeCoNi-HNTAs and MoS_2/Ni Foam.

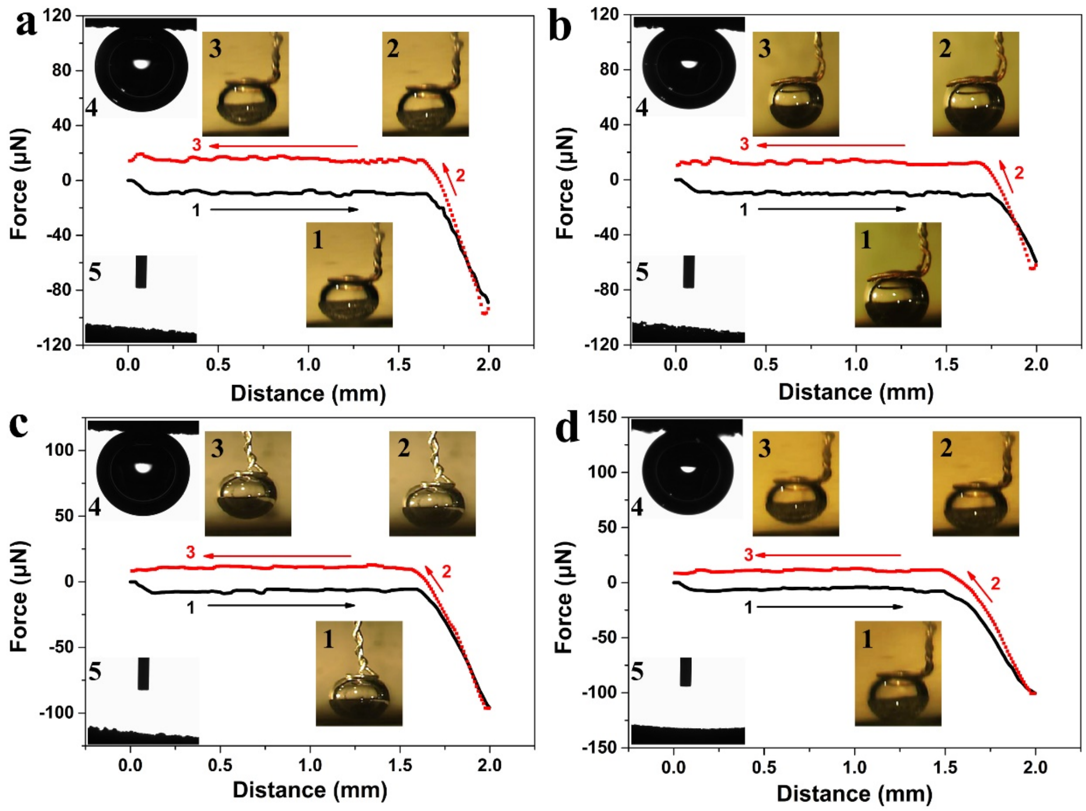

Supplementary Figure 13. Under-electrolyte superaerophobic and superhydrophilic measurements. a-d, Gas bubble adhesive force measurements of CoNi-HNTAs, NiFe-HNSAs, FeCoNi-HNWAs and FeCoNiS-NTAs showing no adhesive force to the bubble on the surface of the four electrodes. The insets 1-3 show the bubble adhesive force measurement process and no distinct bubble deformation can be observed in this process, further illustrating the under-water superaerophobicity of the four samples. For the bubble contact angles under water (insets 4), they were measured to be 155.9° ± 3.1°, 151.0° ± 2.9°, 153.1° ± 3.0° and 152.9° ± 3.4° respectively. Insets 5 display the superhydrophilicity of the four contrastive electrodes because KOH solution droplets could not be captured.

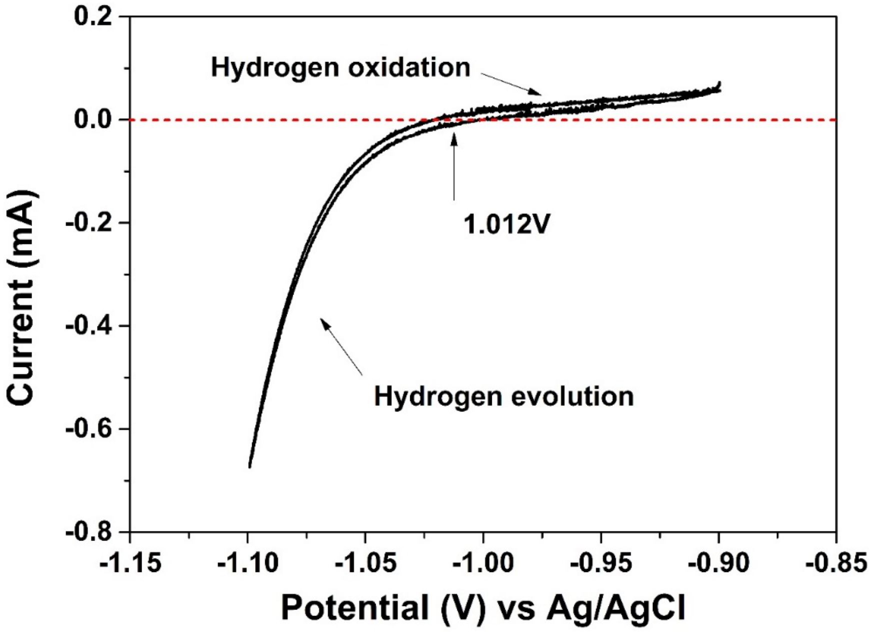

Supplementary Figure 14. RHE calibration. RHE calibration plot. In 1 M KOH, E (RHE) = E (SCE) + 1.012 V.

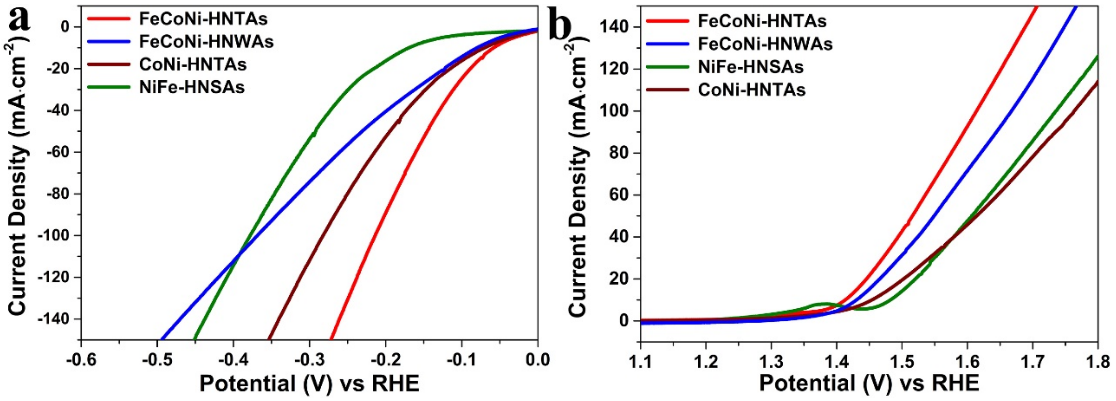

Supplementary Figure 15. The polarization ability for HER and OER. a,**b**, The comparison of polarization curves for HER and OER respectively of as-synthesized nanoarray electrodes measured at a scan rate of 1 mV·s^{-1} in 1 M KOH solution.

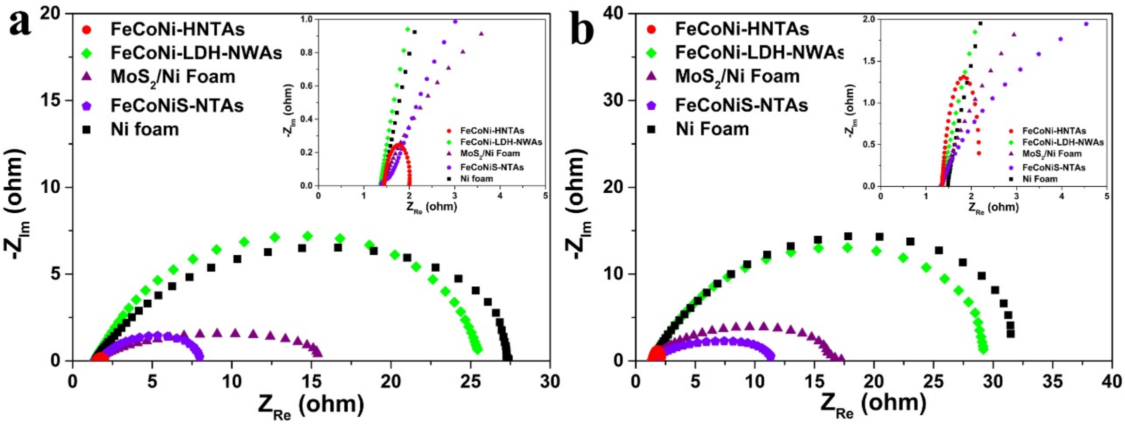

Supplementary Figure 16. The resistances for HER and OER. a,**b**, EIS Nyquist plots of FeCoNi-HNTAs, FeCoNiS-NTAs, FeCoNi-LDH-NWAs, MoS_2/Ni Foam and bare Ni foam at the open circuit voltage for HER and OER respectively. Insets are detailed illustrations for showing the much smaller resistance of FeCoNi-HNTAs.

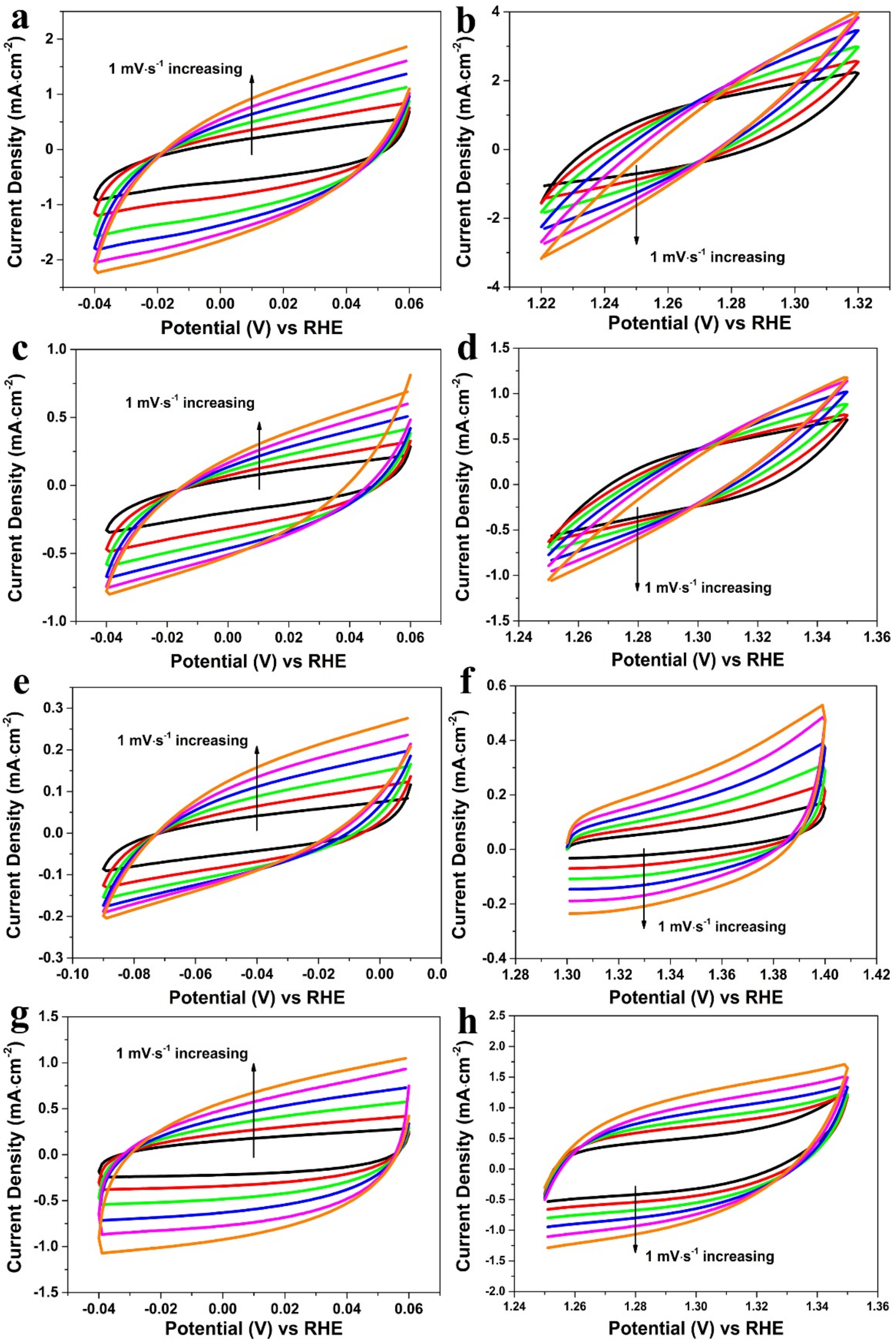

Supplementary Figure 17. Non-Faradaic scan for double-layer capacitance. a, **c**, **e, g,** and **b**, **d**, **f, h,** Cyclic voltammetry for HER and OER respectively at the scan rates of 1, 2, 3, 4, 5, 6 mVs^{-1} in the range of no Faradaic processes for measuring C_{dl} of FeCoNi-HNTAs, MoS_2/Ni Foam and FeCoNi-LDH-NWAs and FeCoNiS-NTAs.

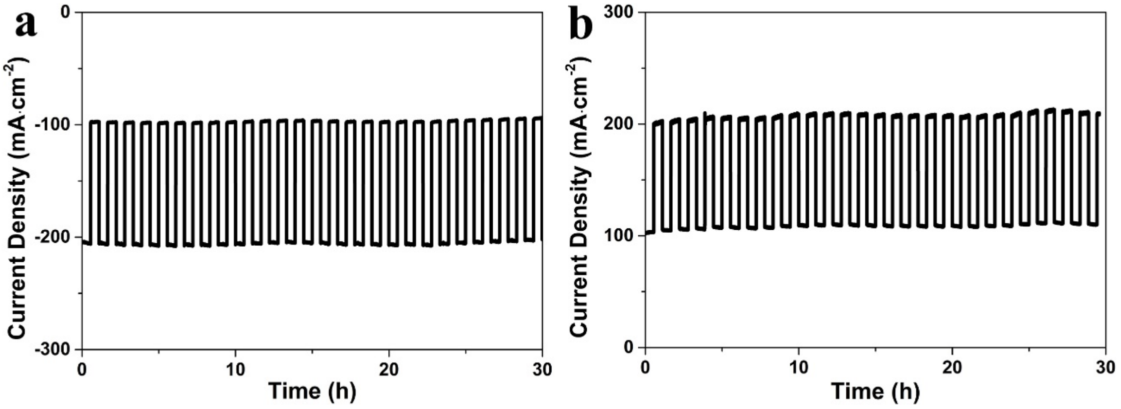

Supplementary Figure 18. The structural recoverability of FeCoNi-HNTAs during HER and OER. a,**b**, Periodic galvanic pulses between two current density of 100 mA cm^{-2} and 200 mA cm^{-2} during 30 h for HER and OER respectively recorded on FeCoNi-HNTAs showing splendid structural recoverability.

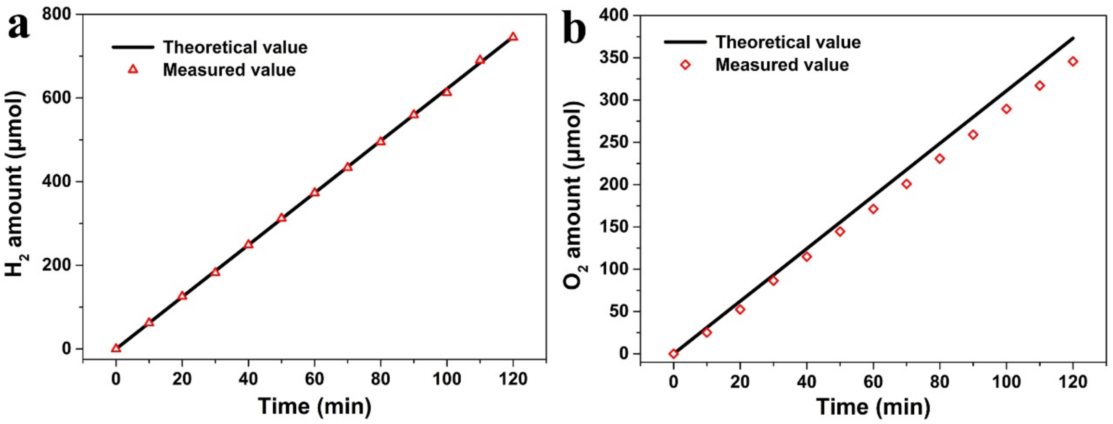

Supplementary Figure 19. The Faradaic efficiency of FeCoNi-HNTAs for HER and OER. a,**b**, Electrocatalytic Faradaic efficiencies of HER and OER respectively over FeCoNi-HNTAs at a current density of 20 mA·cm^{-2} measured for 120 min.

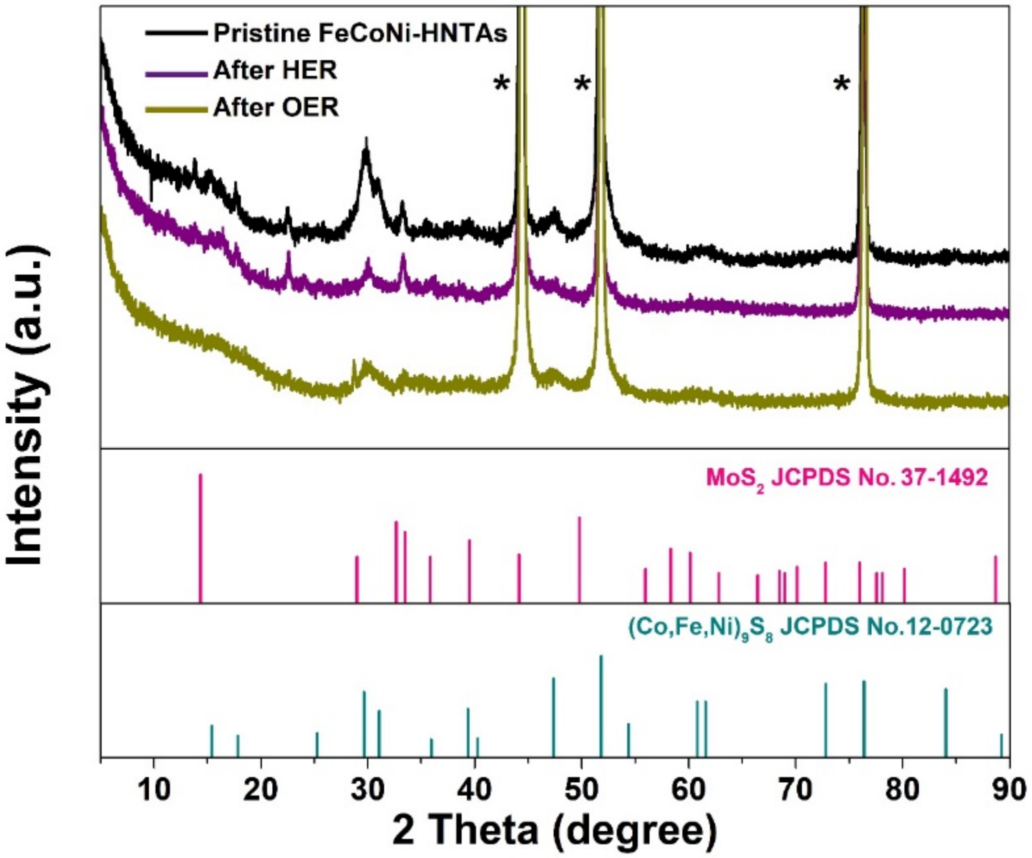

Supplementary Figure 20. The crystalline structures of FeCoNi-HNTAs after HER and OER. XRD pattern of FeCoNi-HNTAs after 1000 cycles of HER and OER. The pink lines present the standard MoS_2 (JCPDS No. 37-1492) peaks, the dark cyan lines demonstrate the standard $(Co,Fe,Ni)_9S_8$ (JCPDS No. 12-0723) peaks. The three peaks of the samples marked by stars represent nickel metal from the Ni foam.

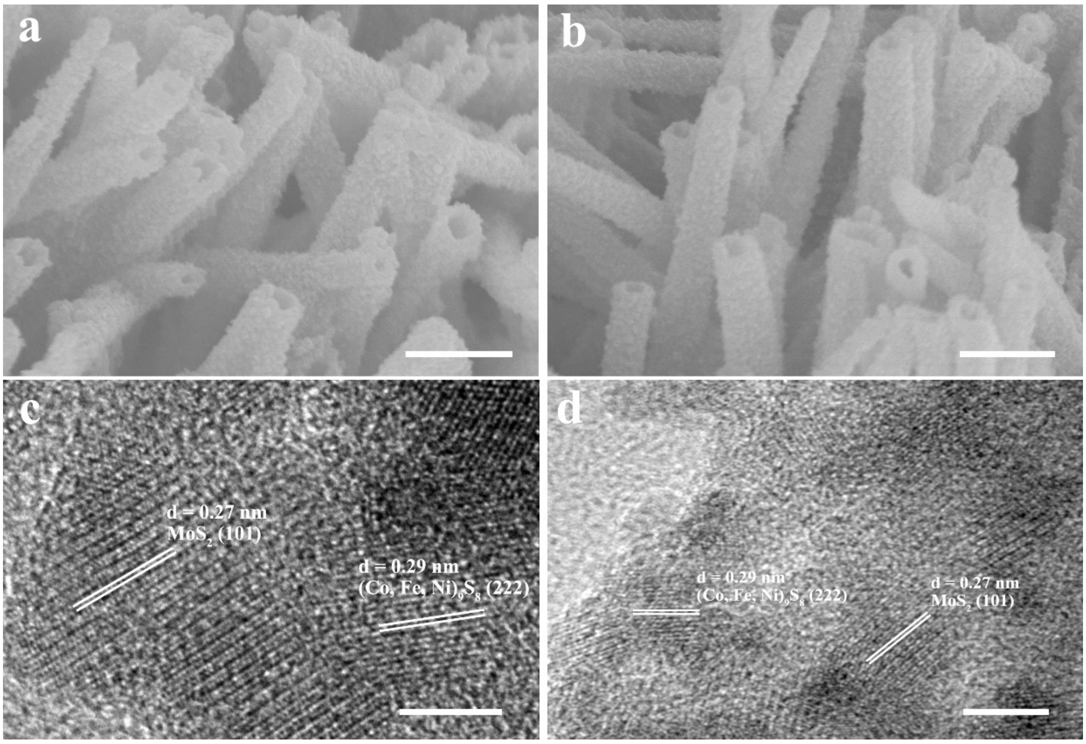

Supplementary Figure 21. The morphology and structure of FeCoNi-HNTAs after HER and OER. a,**b**, and **c**,**d**, FESEM and HRTEM images of FeCoNi-HNTAs after 1000 HER and OER cycles respectively. Scale bar: **a**,**b**, 500 nm; **c**,**d**, 5 nm.

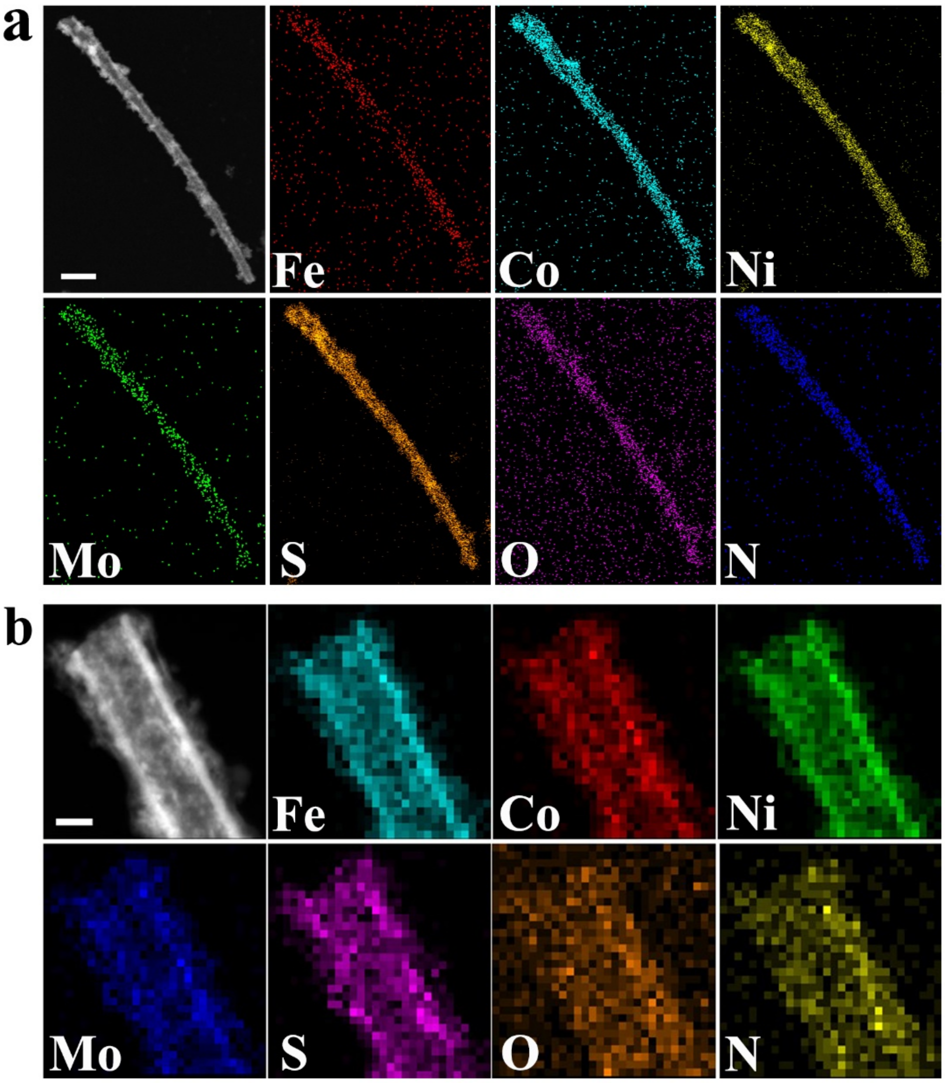

Supplementary Figure 22. The elemental compositions of FeCoNi-HNTAs after HER and OER. a,**b**, STEM and EDX mapping spectra of FeCoNi-HNTAs after 1000 HER and OER cycles respectively showing the uniform distribution of the seven elements. Scale bars: **a**, 200 nm; **b**, 100 nm.

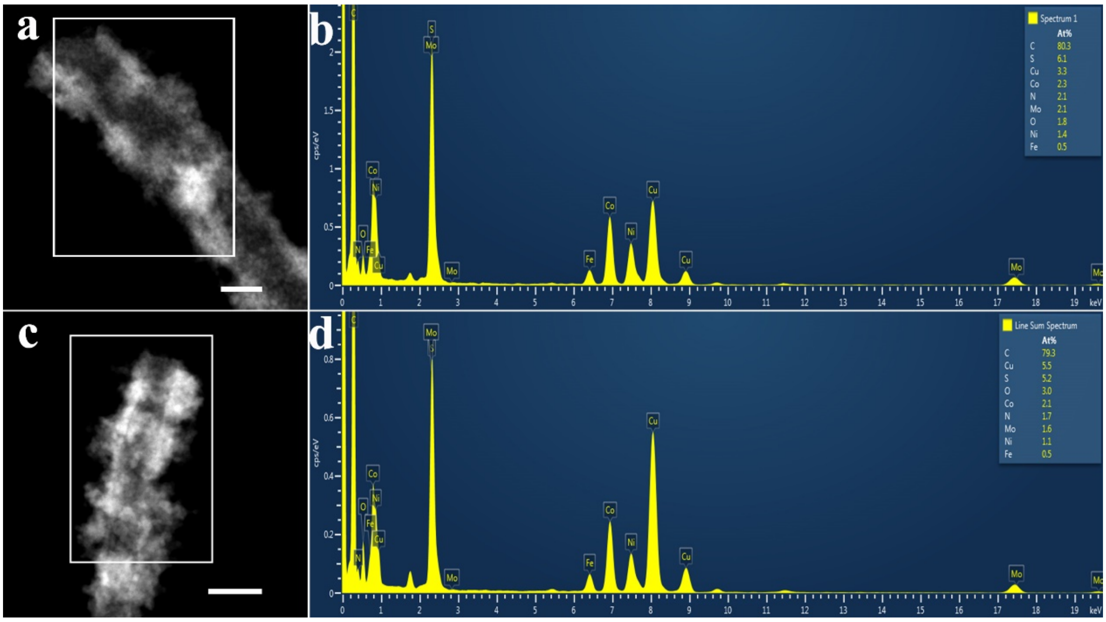

Supplementary Figure 23. The elemental ratios in FeCoNi-HNTAs after HER and OER. a, STEM image of FeCoNi-HNTAs after 1000 HER cycles. **b**, The corresponding EDX spectra of the area marked in **a**. **c**, STEM image of FeCoNi-HNTAs after 1000 OER cycles. Scale bar, **a**, 100 nm; **c**, 200 nm. **d**, The corresponding EDX spectra of the area marked in **c**.

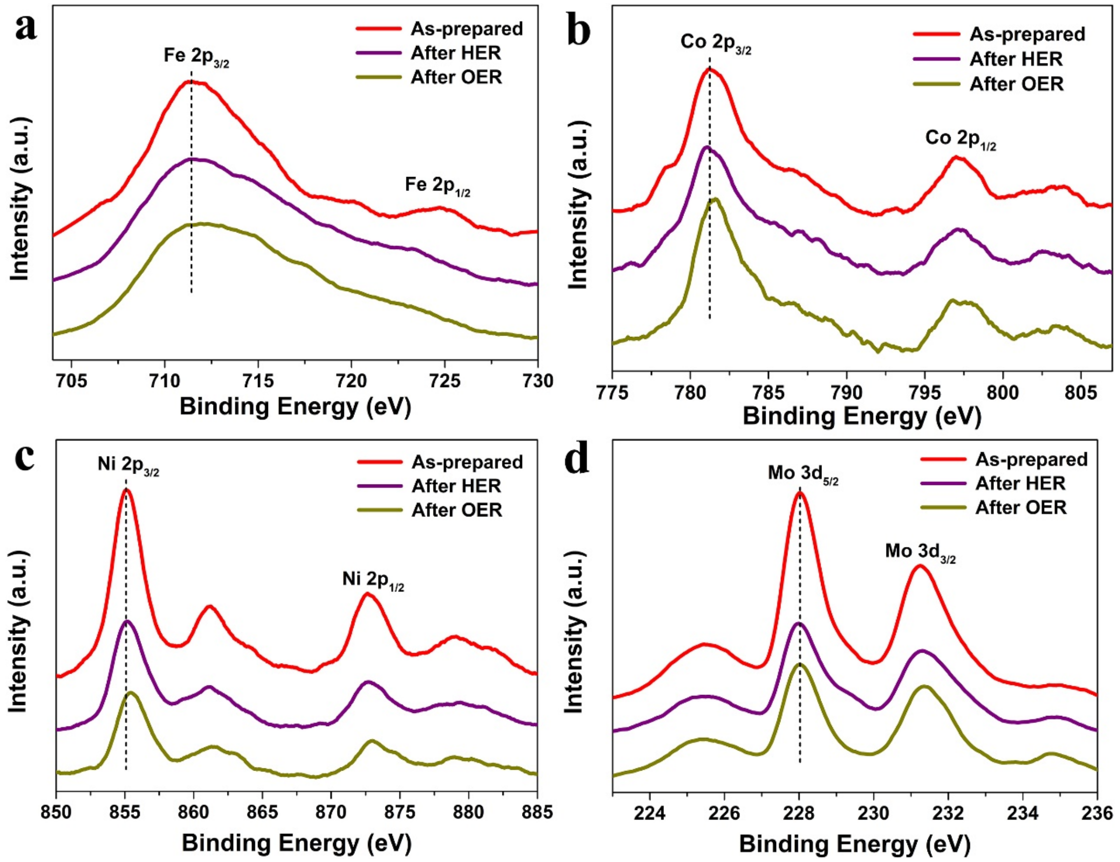

Supplementary Figure 24. The elemental valence states of FeCoNi-HNTAs after HER and OER. a-d, The smoothing XPS spectra showing comparisons among the binding energies of Fe, Co, Ni and Mo in FeCoNi-HNTAs before and after 1000 HER and OER cycles respectively.

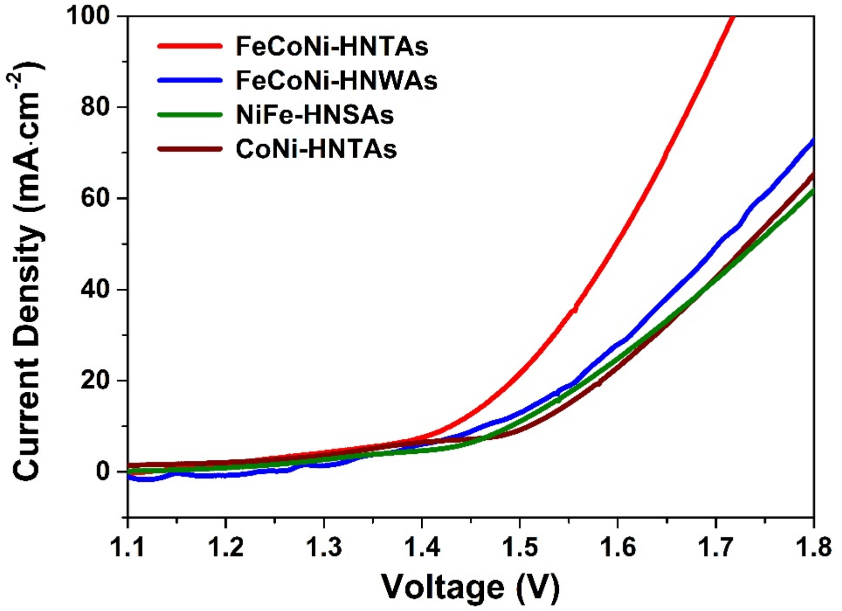

Supplementary Figure 25. The polarization ability for OWS. The comparison of polarization curves for overall water splitting of as-synthesized nanoarray electrodes measured at a scan rate of 1 $mV \cdot s^{-1}$ in 1 M KOH solution.

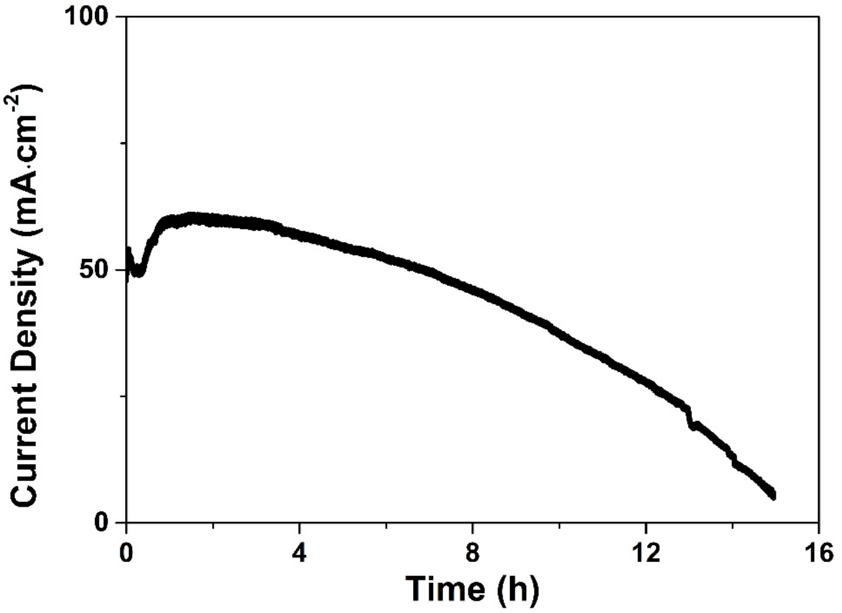

Supplementary Figure 26. The stability of commercial IrO_2/C-Pt/C for OWS. Chronoamperometric curve of overall water splitting over commercial IrO_2/C-Pt/C supported by Ni foam at a constant cell voltage of 1.8 V.

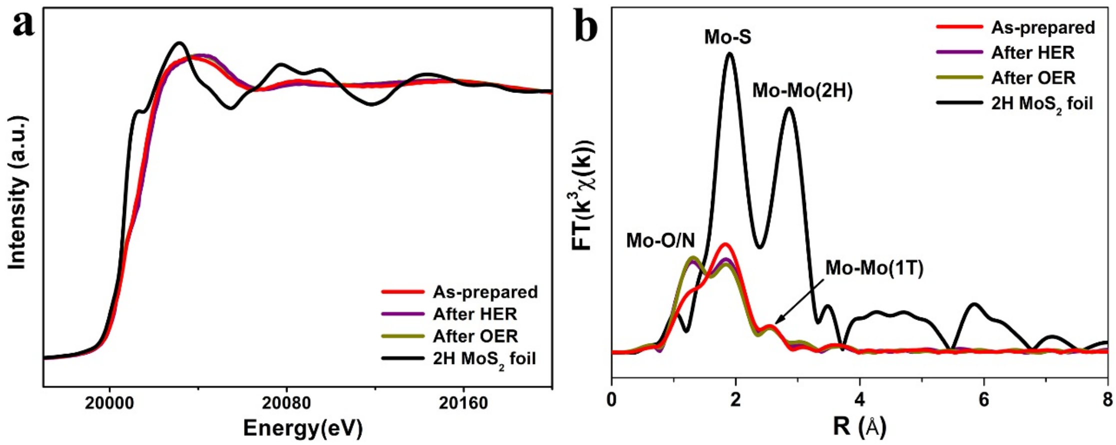

Supplementary Figure 27. The electrochemical stability of 1T' MoS_2. a,**b**, The normalized *ex situ* XANES spectra from EXAFS and the corresponding k^3-weighted FT profile in R-space at Mo K-edge of FeCoNi-HNTAs at as-prepared, after HER and after OER states.

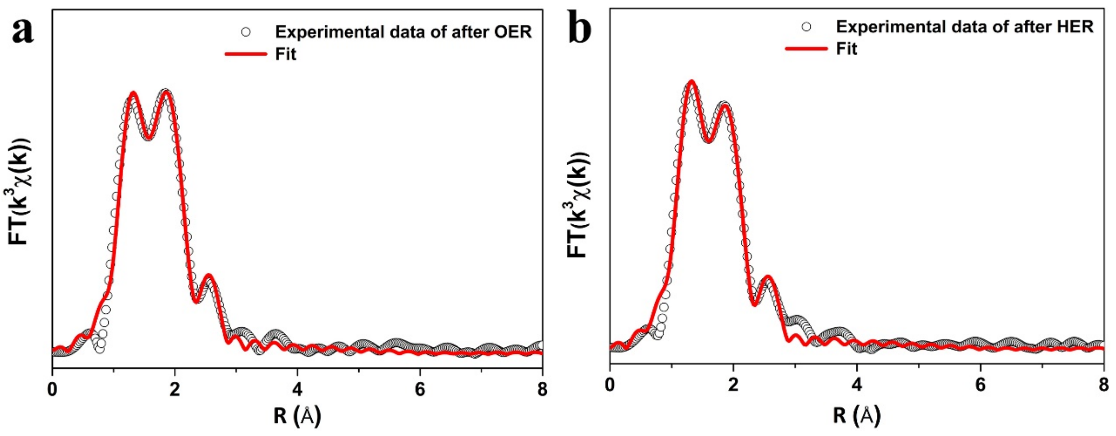

Supplementary Figure 28. The fitting data of *ex situ* EXAFS after HER and OER. **a**,**b**, Comparison between experimental data and the fitting curves of FT profiles from *ex situ* EXAFS for MoS_2 in FeCoNi-HNTAs after HER and OER at Mo K-edge respectively.

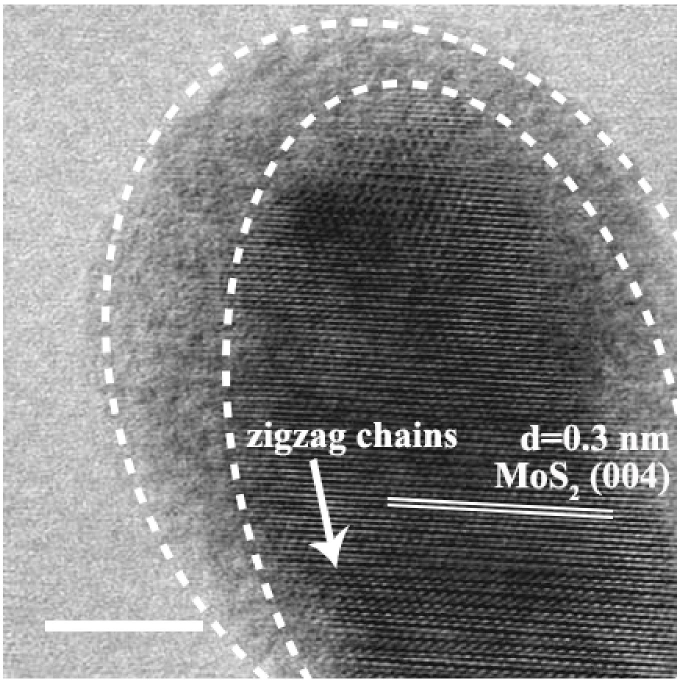

Supplementary Figure 29. The structure of 1T' MoS_2 after OER. HRTEM image showing 1T' MoS_2 with the protection of amorphous component layer after 1000 OER cycles. The zigzag chain is marked by the arrow and the amorphous part is located between the two semi-round dash circles. Integrated with the XRD pattern (Supplementary Fig. 18), the (004) lattice fringes of MoS_2 with the inter-planar distance of 0.3 nm are also demonstrated. Scale bar, 5 nm.

Supplementary Tables

Supplementary Table 1. The fitting results of EXAFS data. Structural parameters of coordination numbers (N), the bond lengths, the Debye-Waller factors (σ^2) for Mo atoms fitted from *ex situ* EXAFS data. Error bounds (accuracies) were estimated as N, ±5%; R, ±1%; σ^2, ±1%.

	Path	N (coordination number)	Bond length	$\sigma^2 (10^{-3}$ Å$^2)$
As-prepared FeCoNi-HNTAs	Mo-O/N	0.9	1.77	1.3
	Mo-S	3.4	2.33	4.9
	Mo-Mo	1.8	2.79	6.3
After HER	Mo-O	1.6	1.76	1.0
	Mo-S	2.6	2.31	6.3
	Mo-Mo	1.2	2.77	5.8
After OER	Mo-O	1.7	1.74	1.4
	Mo-S	2.4	2.30	6.7
	Mo-Mo	1.0	2.79	6.0
2H MoS_2 foil	Mo-S	6	2.41	3.2
	Mo-Mo	6	3.16	3.6

Supplementary Table 2. The data of under-electrolyte superaerophobic and superhydrophilic measurements. The values of adhesive force, bubble contact angle, the advancing angle, the receding angle, the corresponding contact angle hysteresis and electrolyte droplets contact angle for FeCoNi-HNTAs, FeCoNi-LDH-NWAs, MoS_2/Ni Foam, bare Ni foam, CoNi-HNTAs, NiFe-HNSAs, FeCoNi-HNWAs and FeCoNiS-NTAs.

Samples	adhesive force (μN)	bubble contact angle (°)	advancing angle (°)	receding angle (°)	hysteresis (°)	electrolyte droplets contact angle (°)
FeCoNi-HNTAs	0	171.0	172.2	169.7	2.5	0
FeCoNi-LDH-NWAs	0	156.5	157.8	154.6	3.2	0
MoS_2/Ni Foam	10	146.2	149.7	141.1	8.6	0
bare Ni foam	17	143.7	148.6	137.2	11.4	0
CoNi-HNTAs	0	155.9	157.3	154.6	2.7	0
NiFe-HNSAs	0	151.0	153.5	148.7	4.8	0
FeCoNi-HNWAs	0	153.1	155.0	152.1	2.9	0
FeCoNiS-NTAs	0	152.9	155.2	151.8	3.4	0

Supplementary Table 3. The data of Faradaic efficiency measurements. The theoretical values, measured values and Faradaic efficiencies for HER and OER at fixed periods over FeCoNi-HNTAs. The average values of Faradaic efficiency for HER and OER are 99.7% and 90.9% respectively.

	HER			OER		
Time (min)	Theoretical value (μmol)	Measured value (μmol)	Faradaic efficiency (%)	Theoretical value (μmol)	Measured value (μmol)	Faradaic efficiency (%)
10	62.19	62.1	99.9	31.1	25.1	80.7
20	124.37	125.4	100.8	62.18	52.4	84.3
30	186.56	181.9	97.5	93.18	86.4	92.7
40	248.74	248.8	100.0	124.38	114.9	92.4
50	310.93	312.1	100.4	155.46	144.5	92.9
60	373.11	372.9	99.9	186.56	171.3	91.8
70	435.3	433.5	99.6	217.66	200.9	92.3
80	497.49	494.8	99.5	248.74	230.8	92.8
90	559.67	559.4	100.0	279.84	259.2	92.6
100	621.86	612.6	98.5	310.92	289.6	93.1
110	684.04	690.2	100.9	342.02	317.1	92.7
120	746.23	744.8	99.8	373.12	345.7	92.7

Supplementary Note 1.

The as-prepared sample for *ex situ* EXAFS measurements was aging for one month at ambient environment to show oxidation resistance from air. The After-HER and After-OER samples for *ex situ* EXAFS measurements were operated by galvanostatic scan at 20 $mAcm^{-2}$ for 12 h before EXAFS measurements respectively. And the samples for *in situ* EXAFS measurements are fresh to demonstrate the effects of electrocatalytic processes on 1T' MoS_2 and eliminate disturbance of air oxidation.
