# Supplementary material for: Systematic design of superaerophobic nanotube-array electrode comprised of transition-metal sulfides for overall water splitting
Source: Nat Commun. 2018 Jun 22;9:2452. doi: 10.1038/s41467-018-04888-0 (PMC6014975; doi:10.1038/s41467-018-04888-0)
Supplement: Supplementary file 3 — Description of Additional Supplementary Files [file 41467_2018_4888_MOESM3_ESM.pdf]

Description of Additional Supplementary Files

File Name: Supplementary Movie 1
Description: **The overall water splitting process.** The continuous polarization for OWS via chronoamperometric method at a cell voltage of 1.55 V for about 1.5 min. The as-formed H_2 and O_2 bubbles released continuously from the left and the right electrode respectively.
